# An Ultrasound Imaging Repository of the Pediatric Pancreas: Quantitative Image Feature Analysis and Preliminary Feasibility Assessment of Image-Guided Therapeutic Ultrasound Diabetes Treatment

**DOI:** 10.7759/cureus.90394

**Published:** 2025-08-18

**Authors:** Gantt W Meredith, Pavel S Yarmolenko, Karun V Sharma, Vesna Zderic

**Affiliations:** 1 Biomedical Engineering, George Washington University, Washington, DC, USA; 2 Interventional Radiology, Children's National Hospital, Washington, DC, USA; 3 Interventional Radiology, Sheikh Zayed Institute for Pediatric Surgical Innovation, Washington, DC, USA

**Keywords:** abdominal ultrasound, diabetes mellitus, image feature extraction, pancreatic imaging, therapeutic ultrasound

## Abstract

Introduction

There is a lack of comprehensive pediatric pancreatic imaging repositories, which limits ultrasound (US)-based diagnosis and development of potential interventions, such as TUS stimulation for insulin release in diabetes mellitus (DM) therapy. In light of this, a US imaging repository of the pancreas was created to assess diagnostic and therapeutic ultrasound (TUS) feasibility in pediatric patients with DM. US imaging features were extracted to distinguish between DM and non-DM pancreatic tissue, informing preliminary evaluation of patient-specific TUS models.

Methods

Clinical records of pediatric patients were reviewed for the presence of pancreatic US imaging and DM diagnosis. Abdominal US images were reviewed qualitatively with a board-certified radiologist for the presence of the pancreas. Upon pancreas identification, image processing was used to extract quantitative features from the pancreas to compare between DM and non-DM patients. Statistical evaluation was performed via Student's t-test, Spearman's correlation (ρ), mutual information (MI) score, and univariate logistic regression to assess feature predictive performance. Receiver operating characteristic (ROC) curves and area under the curve (AUC) values were calculated for each feature, with 95% confidence intervals (CIs) estimated via stratified bootstrapping (n = 2,000 resamples) to quantify variability. Preliminary TUS modeling from the analyzed images was performed to confirm thermal safety effects of potential TUS protocols for DM therapy. One Food and Drug Administration (FDA)-approved high-intensity therapeutic ultrasound software and one previously published TUS model were validated against an in-house Python TUS simulator informed by patient-specific images. Root mean square error (RMSE) was calculated between the thermal outputs generated by each simulation software.

Results

A US repository for 11 DM and 11 non-DM pediatric patients was created. Imaging features such as entropy (Spearman coefficient (ρ): -0.57, p<0.001, MI score: 0.27), and Centroid Y (ρ: -0.61, p<0.001, MI score: 0.31) support distinctive features in DM patients. Univariate logistic regression analysis identified LBP energy (AUC = 0.79, 95% CI: 0.70-0.87), Haralick inverse difference moment (AUC = 0.78, 95% CI: 0.69-0.86), and Haralick angular second moment (AUC = 0.78, 95% CI: 0.69-0.86) as high performing predictors of DM status. Bootstrapped CIs highlighted stable predictive performance for these texture uniformity features, whereas other descriptors, such as Centroid X and gradient magnitude, showed lower discrimination with CIs falling below the threshold for random predictive performance (0.5). For TUS-mediated insulin release, focused and unfocused simulations confirm safe targeting of pancreatic tissue accessible to treatment with TUS at 1 MHz, 5W/cm^2^.

Conclusions

A US imaging repository of the pediatric pancreas was created as patient-specific source material for diabetic imaging feature analysis and potential TUS for DM therapy. Imaging features revealed relationships between DM pathology and tissue visibly imperceivable to trained radiologists.

## Introduction

Diabetes mellitus (DM) is a metabolic disease primarily affecting insulin release and blood-glucose homeostasis. Impacting more than 37 million Americans [1], DM incidence is on the rise globally. Sources published from 1990 to 2018 across 138 countries support the gradual increase in DM on a global scale [1]. As of 2019, 463 million people globally have a diagnosis of DM. By the year 2030, that number is expected to rise to 578 million and will continue to rise to an estimated 700 million by 2045. By that same year, it is predicted that diabetes will cost the global economy 850 billion USD [1]. The expected increase in disease onset and disease cost makes diabetes and its primary organ, the pancreas, a focus of medical diagnosis and therapy.

In the pediatric population, DM is growing rapidly, doubling from 2002 to 2018 in the United States [2]. According to the Centers for Disease Control and Prevention (CDC), as of 2018, there were 18,000 type 1 diabetic (T1DM) and 5,000 type 2 diabetic (T2DM) cases documented in children. At the current rate of T2DM increase, by the year 2060, it is projected that there will be a 700% increase compared to the current prevalence of T2DM in pediatric populations, compared to a 3.4% increase in T1DM [3]. Furthermore, after the coronavirus disease 2019 (COVID-19) pandemic, new T2DM cases in pediatric patients witnessed an increase of over 60% [4], underscoring the need to focus on T2DM diagnosis and treatments in younger populations.

Although typically detected through blood tests, such as blood glucose and hemoglobin A1c (HbA1c), imaging detection of T2DM, specifically, has been documented through imaging feature analysis. Imaging features, or quantitative measurements of pixel relationships within an image, were able to successfully differentiate between T2DM and non-DM pancreatic tissue in a sample size of 45 T2DM and 33 non-DM patients [5]. From cross-sectional imaging, the adult diabetic pancreas is characterized by serrated edges, a decrease in total volume, and a decrease in tissue homogeneity [6-8].

CT and MRI imaging capture a more holistic imaging window than ultrasound (US) imaging, offering clinicians access to explore abdominal anatomy. However, shorter imaging time, lack of ionizing radiation exposure, greater physical comfort, and lower cost provide strong motivation to further develop US as a potential diagnostic modality in pediatric populations [9,10]. Despite the absence of existing data on US-based imaging of the pediatric diabetic pancreas, visualization of the pancreas is safe and practical with transabdominal US. Standard abdominal US imaging protocols in pediatric hospitals often include images of the pancreas [11], but the utility of transabdominal US for the DM pediatric pancreas has yet to be investigated.

Ultrasound has the unique potential to diagnose and characterize, and with future validation, provide therapy for patients with DM through facilitated insulin release from pancreatic beta cells. This study aims to create the first transabdominal US imaging repository of the pediatric pancreas in patients with and without diabetes. Previous evaluation of the diabetic pancreas has been restricted to cross-sectional imaging [12] and adult populations [13]. US imaging has yet to be evaluated for pediatric DM patients. In our work, pancreatic masks were extracted from radiologist-defined regions of interest (ROIs) to both identify and isolate the pancreas for further analysis. Our analysis investigated imaging features of the diabetic pancreas [5-8, 12,13], including texture-based features, morphology, and depth within the body. These features can inform the selection of US-based imaging biomarkers, improving diagnostic capabilities from US imaging for DM. Further, this work includes a preliminary assessment of therapeutic US (TUS) using pre-validated sonication parameters [14] to inform patient-specific models based on patient data from our imaging repository.

Objectives

The primary purpose of this work is to provide a pediatric diabetic US imaging repository, something that currently does not exist. From this repository, a quantitative assessment of differences between diabetic and nondiabetic images is performed to inform future clinicians' detection of DM from US images. To further expand the clinical toolbox for DM, preliminary therapeutic ultrasound modeling informed by these same B-mode US images suggests that previous *in silico* models support therapeutic US as a treatment for DM [14]. In essence, this work provides further groundwork for diabetic detection and therapy, informing patient-specific clinical models for the amelioration of DM.

## Materials and methods

Pancreatic ultrasound image acquisition

**Figure 1:**
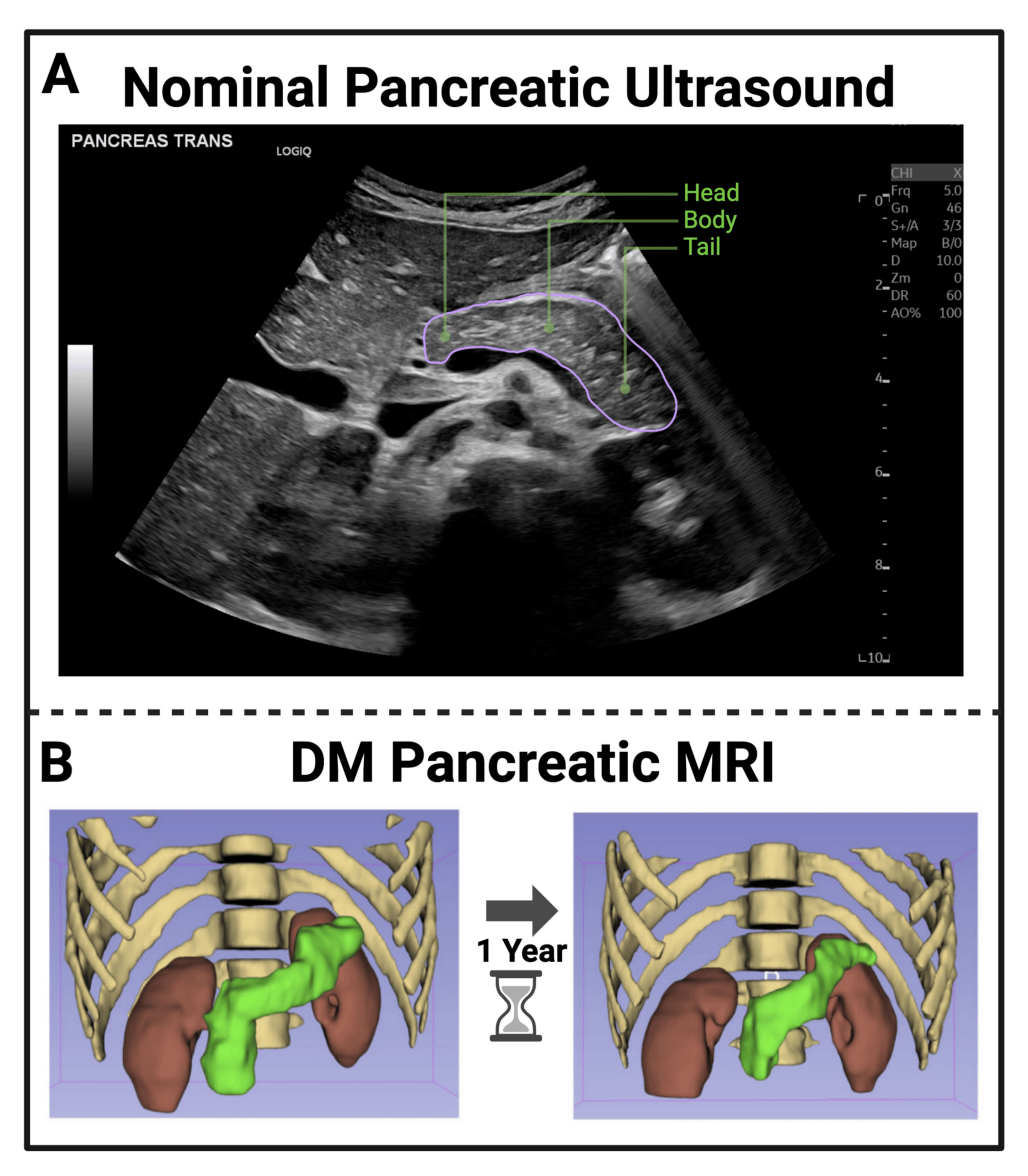
A: Nominal B-mode ultrasound image of a six-year-old male pancreas. B: Cross-sectional imaging illustration of T1DM volume loss from a 19-year-old male via MRI While standard cross-sectional imaging (MRI) demonstrates volume and size reduction in DM pancreas imaging (B), even with ideal US windows of the pancreas (A), similar visualization proves more difficult for clinicians. The head, body, and tail of the pancreas are highlighted in green, and the pancreas is outlined in purple in the B-mode US image and green in the MRI rendering [7] DM: diabetes mellitus; MRI: magnetic resonance imaging; T1DM: type 1 diabetes mellitus

In this work, B-mode US images were collected and reviewed for the presence of the pancreas and its anatomical subregions (head, body, tail). Figure 1A displays a well-visualized B-mode ultrasound image of the pancreas outlined in purple. This image, acquired on the same GE LOGIQ E10 system as each B-mode US image in this work’s repository, is representative of the entire pancreas. The pancreatic head, body, and tail locations are highlighted in green. Figure 1B illustrates a representative 3D rendering of a CT scan obtained from a 19-year-old male one year after a diagnosis of DM [7], highlighting the location, shape, and morphology of the pancreas in green. Given the changes observed in cross-sectional imaging, this work investigated the presence of similar morphological changes via imaging feature analysis of B-mode images similar to those shown in Figure 1A.

Anonymized, pediatric B-mode US images were obtained from patients undergoing abdominal US imaging as part of routine abdominal US scanning at Children’s National Medical Center (Washington, DC). The electronic health records (EHR) were searched over six months for patients with confirmed pancreatic presentation and diabetic status. Complete visualization of the pancreas (head, body, and tail) of 11 DM patients was identified, resulting in the subsequent creation of a non-DM imaging dataset matched for pancreas presentation, age, sex, and date of acquisition. Table 1 presents the results of the pancreas imaging query. Of note, both T1DM and T2DM patients are included in the analysis of DM tissue, as there were not enough T2DM patients for a thorough review. Further, T1DM and T2DM tissues have already been validated to contain similar morphological and textural changes [15]. Once the complete pancreas was identified in US images, the pancreas was outlined in HOROS^TM^ (Horos Project, 2025), a digital imaging and communications in medicine (DICOM) viewing software with annotation tools.

**Table 1:** Patient B-mode ultrasound dataset repository information Table 1 illustrates each patient’s sex, age, diabetic status, basic height and weight information, as well as the number of pancreatic US images confirmed to have pancreatic visualization. From this imaging review, 44 diabetic US images and 46 non-diabetic US images were analyzed in this work BMI: body mass index; DM: diabetes mellitus; US: ultrasound

ID	DM	Age, years	Sex	Height, cm	Weight, kg	BMI	# US images
Patient 1	T2DM	17	M	154	59.1	24.92	2
Patient 2	T2DM	16	M	185.2	147.1	42.89	3
Patient 2	T2DM	10	F	135	27.2	n/a	10
Patient 4	T1DM	13	M	164.8	78.3	28.83	8
Patient 5	T2DM	8	F	131	27.6	16.08	5
Patient 6	T2DM	17	F	180	130	n/a	2
Patient 7	T2DM	15	M	149.4	75.9	34	3
Patient 8	T1DM	13	F	167.5	66.4	23.67	4
Patient 9	T1DM	2	F	81.5	10.9	16.41	2
Patient 10	T1DM	9	F	139.5	65.4	33.61	2
Patient 11	T1DM	13	F	134.5	34.9	19.29	3
Patient 12	N	10	M	140	36.5	18.62	2
Patient 13	N	5	F	113	19.3	15.11	6
Patient 14	N	8	M	134.9	34	n/a	5
Patient 15	N	14	M	168.1	67.4	23.85	3
Patient 16	N	13	F	167.6	55.8	19.86	4
Patient 17	N	13	F	170	60.6	20.97	3
Patient 18	N	13	F	152	37.9	16.4	4
Patient 19	N	18	M	166.5	42.6	n/a	10
Patient 20	N	13	F	156.5	53.6	n/a	3
Patient 21	N	14	F	96.5	50.7	54.44	3
Patient 22	N	17	F	146.5	58.7	25.95	3

Pancreatic ultrasound image processing and image feature extraction

The head, body, and tail of the pancreas were manually outlined in each B-mode image and confirmed with a board-certified radiologist. Binarized masks of the pancreas were created for pancreas isolation in downstream a) imaging feature statistical comparison and b) preliminary TUS modeling. Image features were extracted via an in-house Python script, PyTU. PyTU utilized validated Python packages for image processing, including NumPy, Pandas, Matplotlib, SciPy, and Scikit-image [16-20]. A table in the next section outlines the imaging features analyzed in this work, incorporating previous observations of changes in pancreatic morphology. It also presents novel features such as the scale-invariant feature transform (SIFT). Keypoints include the transformations of image data into scale-invariant coordinates relative to local features [21]. Many of the features analyzed in this work were Haralick features, which have been applied in pancreatic imaging to differentiate diabetic from non-diabetic tissue [15].

In this work, Haralick features were computed using the open-source Mahotas computer vision library [22], enabling reproducible and standardized feature extraction. Haralick features compare the relative values and flux between pixels within the gray-level co-occurrence matrix (GLCM) for textural comparison. This matrix captures spatial relationships between pixel intensities, revealing tissue-level changes within the pancreas. US, characterized by echogenicity and speckling, is particularly subject to textural feature analysis techniques incorporating GLCM-based features [23] and local binary patterns (LBPs) [24]. These features, in conjunction with basic pixel and mask morphology analysis, offer promising building blocks for potential imaging biomarkers of DM. In this work, imaging features were utilized to understand quantifiable differences between DM and non-DM pancreatic tissue.

Statistical analysis of image features informing diabetes mellitus imaging biomarkers

A Student’s t-test was first performed on the feature dataset to remove any initial imaging features with p<0.05 when determining statistical significance. To understand linear and non-linear relationships between each statistically-significant feature and DM, feature correlation, significance, and mutual information (MI) were calculated. Feature correlation was measured using a Spearman’s coefficient (ρ) calculation. Spearman’s coefficient, rho [25], relates continuous feature values to categorical diabetic status, revealing ranked correlation by feature. Feature significance was measured via a two-sample Student’s t-test comparing each feature and DM status. However, these two statistical comparisons assume a linearly distributed dataset; the previous statistical methods fail to capture both linear and nonlinear relationships within the dataset.

By calculating MI scores for each feature concerning diabetic status, linear and nonlinear relationships between each feature and diabetic status were explored. An MI score, in this context, is a measure of how much information each feature contains about DM status [26], with higher MI scores yielding features more relevant to DM differentiation. MI measures linear and nonlinear relationships describing the amount of information contained within DM classification for each feature. The combination of t-tests, Pearson’s coefficient, and MI has been previously deployed and validated in fetal US [26]. This work attempts to adopt a similar statistical framework to understand US imaging features as potential US imaging biomarkers for DM.

Imaging feature logistic regression

Following univariate statistical screening, binary logistic regression models were fitted individually for each significant feature to assess its predictive ability for classifying diabetic status (1 = DM, 0 = non-DM). Features were standardized via z-score before logistic regression implementation. Discrimination of each feature was evaluated by calculating the area under the receiver operating characteristic curve (AUC). To quantify uncertainty, stratified bootstrapping (n = 2,000 resamples) was performed, preserving class proportions in each sample. For each bootstrap replicate, the univariate AUC was recalculated, and the 2.5th and 97.5th percentiles of the bootstrap distribution were used to calculate a 95% confidence interval (CI). This logistic regression performed in this work produced a combined performance summary integrating statistical significance (p-values from t-tests) with predictive accuracy and robustness (AUC ± 95% CI). Visualization of the statistical analysis, and the statistical analysis itself, was performed in Python (v3.11) via the open-source software packages of Matplotlib, Seaborn [27], Scikit-learn [28], and Statsmodels [29].

Initial patient-specific therapeutic ultrasound modeling for insulin release

Beyond imaging features for DM diagnosis, this work utilized patient-specific images to inform tissue-specific TUS models for DM therapy. A Food and Drug Administration (FDA)-approved high-intensity therapeutic ultrasound (HITU) simulator was used to model focused TUS acoustic propagation and thermal effect modeling. Limited in grid resolution, transducer type, and deeper anatomical targeting, the HITU simulator was only suitable for modeling patients with pancreases within ~5 cm of the abdominal wall. Therefore, foundational equations from the HITU simulator were adapted within PyTU’s image processing pipeline to create similar TUS models informed by patient-specific B-mode images. Previous *in silico* modeling from this group identified optimal unfocused sonication protocols for pediatric TUS therapy [14]. These sonication parameters and unfocused modeling parameters in OnScale were validated against the results of PyTU, focusing on thermal safety effects compared to unfocused TUS application. Thermal dose in units of cumulative equivalent minutes at 43˚C (CEM43) [30] was calculated for unfocused and focused simulations. Thermal dose is among the more established measures of clinical safety and effects of heating tissues in therapeutic ablation applications [30].

Experimental overview

In summation, this work created a pancreatic imaging repository of B-mode US images from DM and non-DM patients. These images were reviewed by a board-certified radiologist, but qualitative visual differences were not easily discernible between DM and non-DM pancreatic tissue. The lack of qualitative observations prompted quantitative analysis of imaging-based features to assess pancreatic tissue morphology and texture. Statistical analysis was performed to understand which features were most impactful in differentiating between DM and non-DM tissue. To extend the clinical impact of this work’s B-mode US analysis, three TUS simulation software packages were deployed to assess patient-specific focused and unfocused TUS. The abdominal tissues measured from B-mode images that intersected the sonication beam path were included in the TUS models, creating a model with tunable focus depths and tissue properties. With recent advances in DM treatment targeting non-pancreatic regions such as the hepatoportal nerve plexus [31], the development of amenable, patient-specific models informed by patient-specific imaging needs to be explored and developed. This work offers preliminary simulations towards patient-specific models of TUS for insulin release.

## Results

Review of pancreatic ultrasound in a pediatric diabetes mellitus dataset

**Figure 2:**
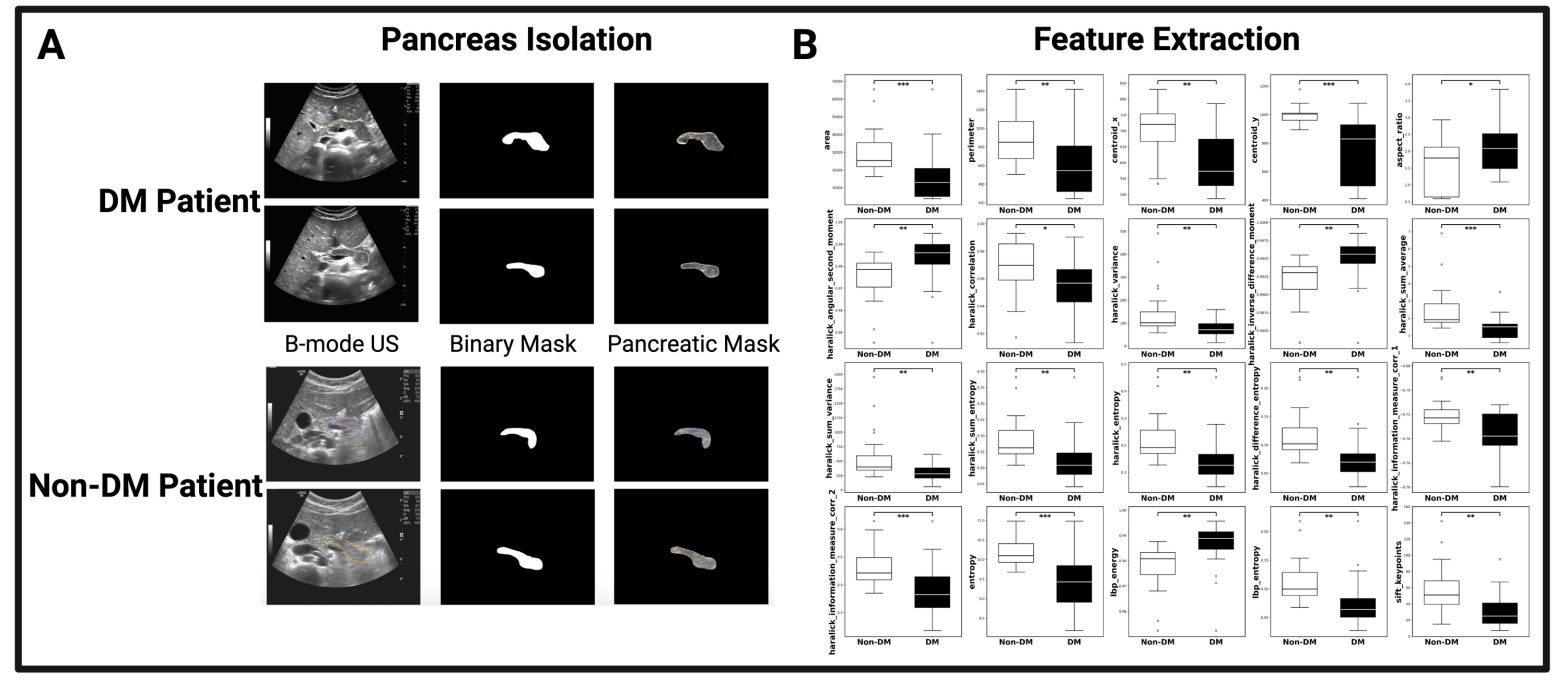
Image processing of the pancreas from B-mode ultrasound images B-mode ultrasound images were analyzed (A), and the pancreas was outlined by a board-certified radiologist. The outline was used to extract a pancreatic mask that was isolated using an open-source Python image processing toolbox. Statistically different features between DM (black) and non-DM (white) patients after a one-way Student’s t-test were visualized via the boxplots in B. From the over 30 textural and morphological features measured, the 20 features with a p-value from an unpaired Student’s t-test of less than 0.05 were included for further analysis (*p<0.05, **p<0.01, *** p<0.001)

**Figure 3:**
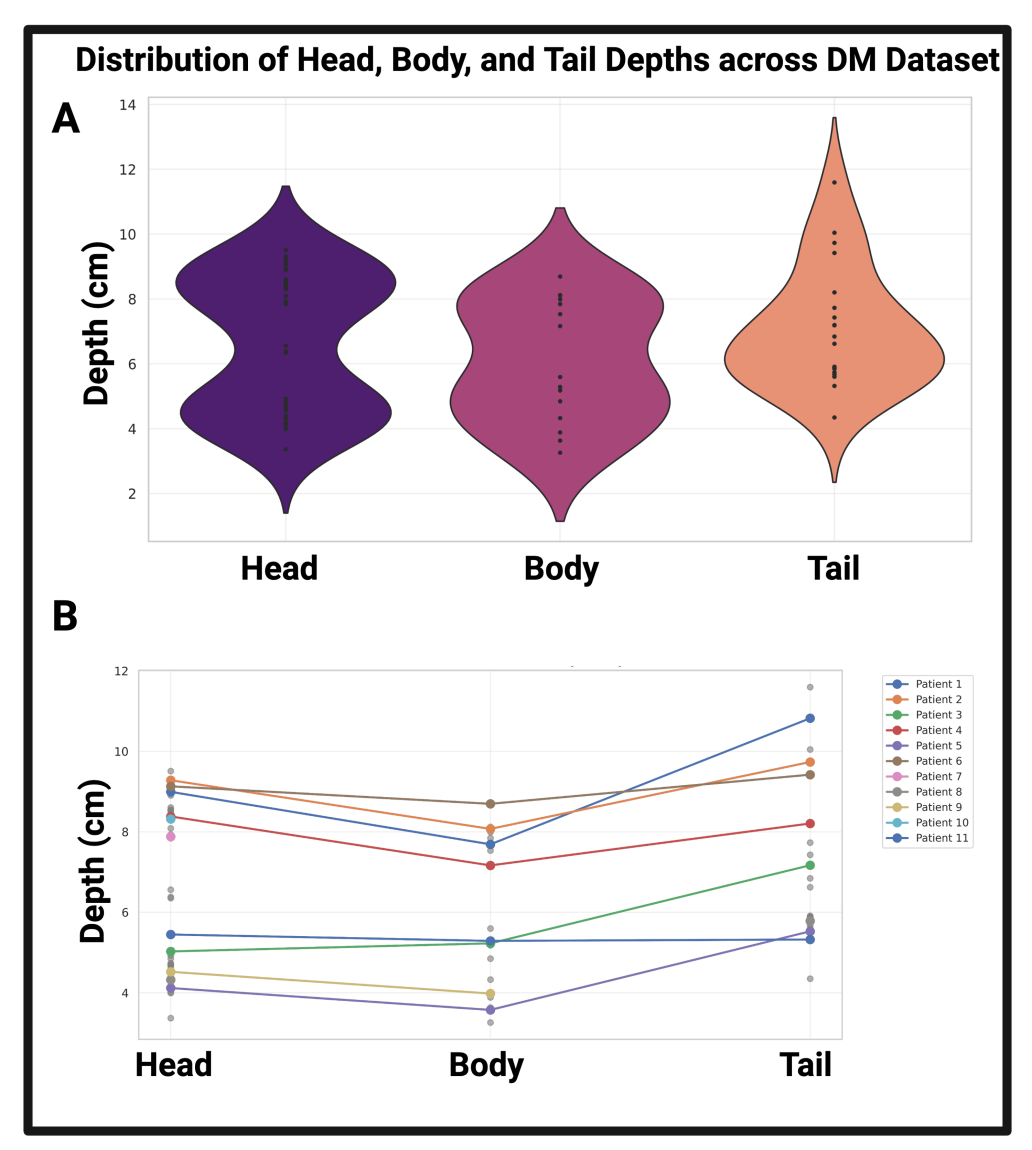
Pancreatic visualization across patients Figure 3A’s violin plots demonstrate the distribution of pancreas identification depths within the overall DM dataset (n = 11 patients, n = 90 images). Figure 3B demonstrates the progression of those depths throughout the pancreatic imaging window, highlighting previous clinical findings of higher visualization of the head and body [13] DM: diabetes mellitus

A nominal pancreatic ultrasound, depicted in Figure 1A, serves as an ideal example of the pancreas and its anatomical subregions. Given the retrospective nature of our review, the visualization of the pancreas from pancreatic ultrasound images was not as complete as the pancreas outlined in Figure 1A. Figure 2’s masks demonstrate an isolation of pancreatic tissue, revealing the morphology of each pancreas without pixel information of surrounding tissues influencing the view of the organ or its further image processing. From the image processing outlined in Figure 2, Figures 3A-3B illustrate the distribution of head, body, and tail depths relative to the patient’s dermis. Figure 3A’s violin plots illustrate the distribution of head, body, and tail depths across patients, highlighting the increased visualization of the head and body as supported by prior imaging review [13]. Figure 3B’s linear progression visualization illustrates the location of each patient’s pancreatic head, body, and tail as each pancreas image was reviewed. If multiple images of one region were observed, all region depths were plotted, but the mean value was used for linear depth progression.

Imaging feature measurements stratified by diabetic and non-diabetic patients

Upon pancreatic imaging review, qualitative differences between DM and non-DM pancreatic tissue (Figure 2) were not visually discernible by a board-certified radiologist. Therefore, a comparative analysis was conducted to identify potential image-based features that significantly differ between DM and non-DM patients. Student, two-sample t-tests for each extracted feature were performed, and only those with statistically significant differences (p<0.05) between DM and non-DM patients were visualized using boxplots in Figure 2B. Only patients whose abdominal scans contained complete pancreatic anatomy (head, body, and tail) were included in this t-test analysis (seven diabetic and seven non-diabetic patients). A total of 48 pancreatic regions (24 from each group of seven patients) were used in the Student's t-test comparison highlighted in Figure 2B. This comparison thus includes 46 degrees of freedom. Table 2 contains the results of the t-test, including degrees of freedom, p-values, and t-values. Of note, features such as Centroid Y (t = 5.16, p<0.001), entropy (t = 4.95, p<0.001), Haralick information measure of correlation 2 (t = 3.85, p<0.001), and Haralick sum entropy (t = 3.21, p = 0.002) displayed a strong separation between diabetic and non-diabetic patients. As another example, aspect ratio (t = -2.06, p = 0.045) demonstrated a more modest effect size, further supporting the hypothesis that both geometric shape and textural features are statistically different between diabetic and non-diabetic patient stratifications.

**Table 2:** Statistically significant features extracted from diabetic and non-diabetic comparison of B-mode ultrasound images Each feature extracted from B-mode ultrasound images is found in this table, with each feature containing a brief description, the relative magnitude (+/-) of the feature value for DM US images, and the degree of that feature’s importance based on a Student's t-test (*p<0.05, **p<0.01, ***p<0.001). T-values, p-values, and degrees of freedom (46) for each feature are listed DM: diabetes mellitus; GLCM: gray-level co-occurrence matrix; LBP: local binary pattern; SIFT: scale-invariant feature transform; US: ultrasound

Feature	Description	T-value	P-value	Degrees of Freedom (df)	+­/- in DM US
Area	Complete area by pixel summation	3.64	p<0.001	46	-***
Perimeter	Path around the pancreas mask	3.48	p = 0.001	46	-**
Centroid_x/y	Geometrical center of the pancreas mask	3.46 / 5.16	p = 0.001 / p<0.001	46 / 46	-**/***
Aspect ratio	Ratio of width to height	-2.06	p = 0.045	46	­+*
Haralick angular second moment	GLCM textural uniformity	-3.02	p = 0.004	46	­+**
Haralick correlation	GLCM pixel similarity by neighbor	2.45	p = 0.018	46	-*
Haralick variance	GLCM texture randomness	3.12	p = 0.003	46	-**
Haralick inverse different moment	GLCM local homogeneity	-3.40	p = 0.001	46	­+**
Haralick sum average	GLCM sum of neighboring pixel intensity	3.71	p<0.001	46	-***
Haralick sum variance	GLCM variance of sum intensities	3.14	p = 0.003	46	-**
Haralick entropy	GLCM variability and disorder	3.27	p = 0.002	46	-**
Haralick sum entropy	GLCM disorder from pixel pairs	3.21	p = 0.002	46	-**
Haralick difference entropy	GLCM randomness of intensity differences	3.46	p = 0.001	46	-**
Haralick information measure correlation 1/2	GLCM mutual information of pixel pairs	3.25 / 3.85	p = 0.002 / p<0.001	46 / 46	-**/***
Entropy	Probability distribution of intensity values	4.95	p<0.001	46	-***
LBP entropy	Local binary pattern capturing texture randomness	3.36	p = 0.002	46	-**
LBP energy	Local binary pattern capturing overall consistency in patterns	-3.25	p = 0.002	46	­+**
SIFT keypoints	Scale-invariant feature transform identifying key features impermeable to augmentation	3.25	p = 0.002	46	-**

**Figure 4:**
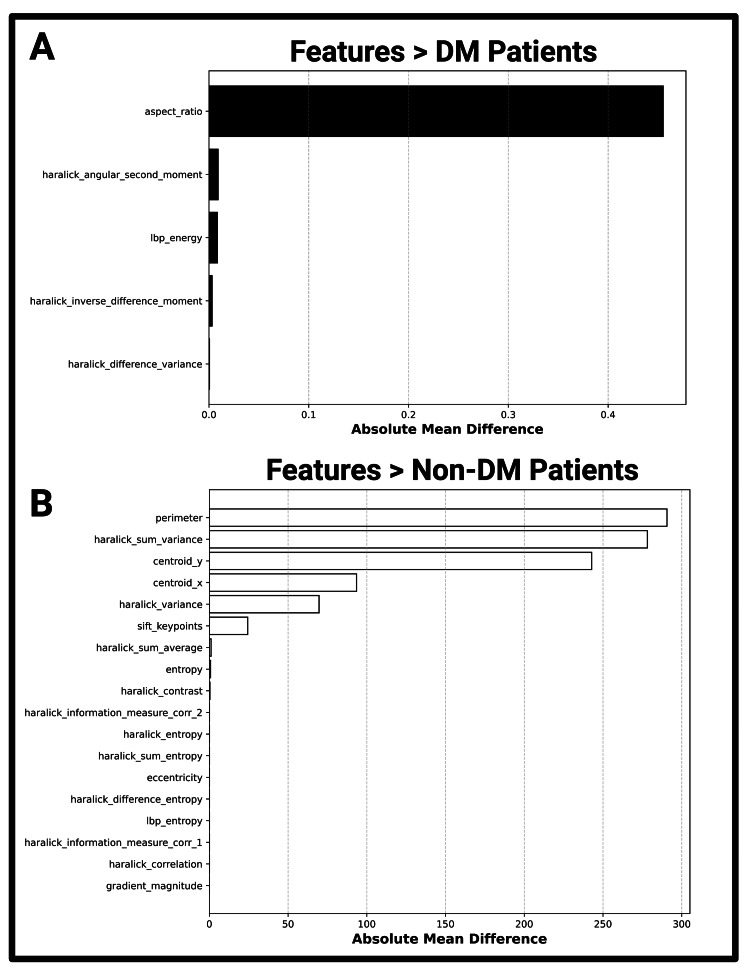
Features with relatively higher values for diabetic and non-diabetic patients Figure 4A illustrates the features with higher values for DM patients calculated via absolute mean difference. Figure 4B illustrates the features with higher values for non-DM patients. The absolute mean difference, or the range-adjusted absolute mean, between DM (44 images) and non-DM (46 images) imaging features represents the initial step towards understanding feature relationships in a DM imaging biomarker context DM: diabetes mellitus

Figures 4A-4B take a closer look at all imaging features (Table 2), regrouping features by relative magnitude and DM status, with the highest relative values for DM and non-DM pancreatic images. Entropy features, both Haralick and conventional, were lower among DM images, challenging conventional literature that cross-sectional imaging entropy increases with DM [5]. Aspect ratio, Haralick moment features, and LBP energy were relatively higher in DM pancreatic imaging analysis. Haralick moment features measure texture uniformity, with higher values indicating more structural integrity and less entropic tissue [23]. Local binary pattern energy measures the textural uniformity of the tissue, with higher values indicating higher textural uniformity. Beyond relative, comparative values, the influence of each feature on DM status is further explored later on.

Imaging feature correlation with diabetes mellitus

**Figure 5:**
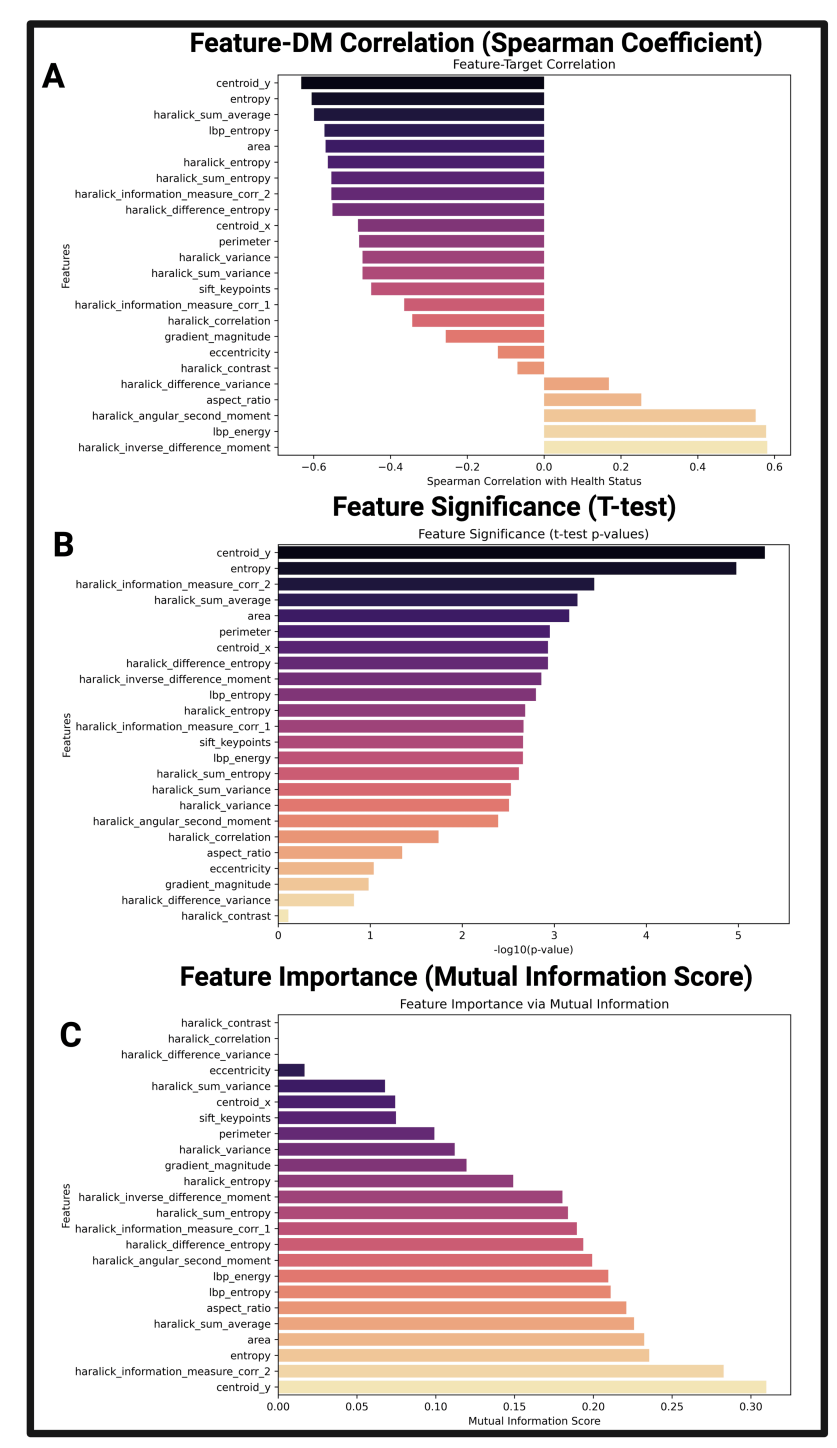
Comparative statistical analysis of pediatric ultrasound features in diabetic and non-diabetic patients Figures 5A-5C illustrate the relationships present within the size, texture, and morphological imaging features extracted from pancreatic B-mode ultrasound images. Figure 5A depicts relationships between feature correlation and diabetic status, with higher Spearman correlation values indicating a high correlation with DM status. Figure 5B depicts the comparative results of a two-tailed t-test assessing the discriminatory potential of these imaging features between DM and non-DM patients. P-values were transformed to a -log(10) scale, with higher values indicating a higher degree of statistical correlation. Figure 5C depicts the results of the mutual information score for each feature: the degree to which the classification (diabetic status) is contained within each feature. Whereas B assumes a normal distribution, C contains assumptions for any feature distribution and any causal relationships (not limited to linear, monotonic, etc.) DM: diabetes mellitus

From this initial review of relative feature values, Figures 5A-5C take a deeper look at the correlation, significance, and importance of each feature with each image’s diabetic classification. Figure 5A reveals features strongly associated with DM as calculated by Spearman’s correlation. Positive and negative values are associated with positive and negative correlation between that feature and DM status. Features such as Haralick sum average (GLCM intensity) and Haralick entropy (GLCM disorder) have negative correlations, suggesting their relative values decrease in DM pancreatic US images. Conversely, features such as Haralick inverse difference moment and LBP energy (both textural uniformity) have a positive correlation with DM, suggesting these values might be higher in the US images of pediatric DM images.

Imaging feature significance and mutual importance with diabetes mellitus

Figure 5B depicts features that are the most significant in explaining DM versus non-DM pediatric US image classification. The results of the two-tailed Student t-test performed were scaled to -log(10) to create a normalized, descending distribution. Features such as Centroid Y (abdominal depth) and Entropy (pixel-level tissue disorder) were statistically significant in this analysis, in concurrence with Figure 5A. Figure 5C takes the analysis one step further by illustrating the MI score of each feature. Via pancreatic ultrasound, features such as Centroid Y and Entropy have a high mutual information score. Features such as eccentricity and aspect ratio, which were lower in relative importance in Figures 5A-5B, have a moderate mutual information score. These observations are addressed further in the discussion section of this work.

Imaging feature logistic regression

**Figure 6:**
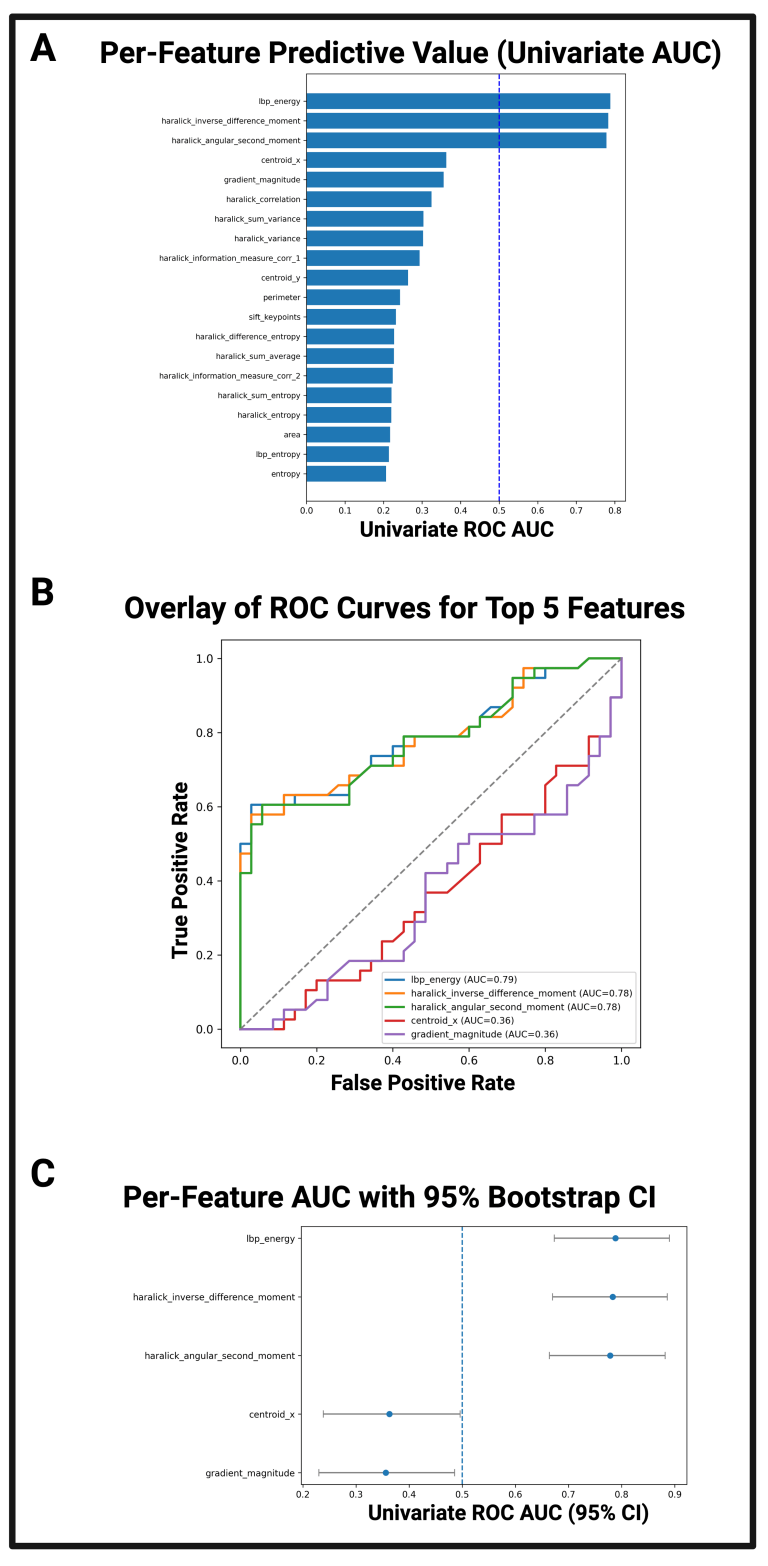
Predictive value of individual imaging features for differentiating DM and non-DM pancreatic ultrasound images Figure 6A: Univariate logistic regression AUC values for all statistically significant features from t-test screening (p<0.05). The dashed vertical line marks chance performance (AUC = 0.5). Figure 6B: Overlay of ROC curves for the five highest-ranked features by AUC. Figure 6C: AUC values for the top five features with 95% confidence intervals calculated from 2,000 stratified bootstrap resamples, illustrating the precision of AUC estimates. LBP energy, Haralick inverse difference moment, and Haralick angular second moment demonstrated both high AUC and narrow CIs, indicating strong, stable discrimination between DM and non-DM pancreatic regions derived from ultrasound images AUC: area under the curve; CI: confidence interval; DM: diabetes mellitus; LBP: local binary pattern; ROC: receiver operating characteristic

Among the statistically significant imaging features, LBP energy displayed the highest univariate predictive value (AUC = 0.79, 95% CI: 0.70-0.87), followed by Haralick inverse difference moment (AUC = 0.78, 95% CI: 0.69-0.86) and Haralick angular second moment (AUC = 0.78, 95% CI: 0.68-0.86) (Figures 6A-C). These three texture uniformity metrics demonstrated both high discrimination and narrow bootstrapped confidence intervals, suggesting stable performance despite a modest sample size of 90 ultrasound images. Conversely, descriptors such as Centroid_x (AUC = 0.36, 95% CI: 0.27-0.46) and Gradient magnitude (AUC = 0.36, 95% CI: 0.26-0.46) displayed lower univariate predictive value, with displayed confidence intervals falling below the value of 0.5 (random classification performance). Figure 6B overlays the top 5 features for logistic regression predictive value. Overlaying the ROC curves highlights a preliminary separation of texture uniformity features and other, weaker features.

Deployment of multiple therapeutic ultrasound models for focused and unfocused modeling of insulin release

**Figure 7:**
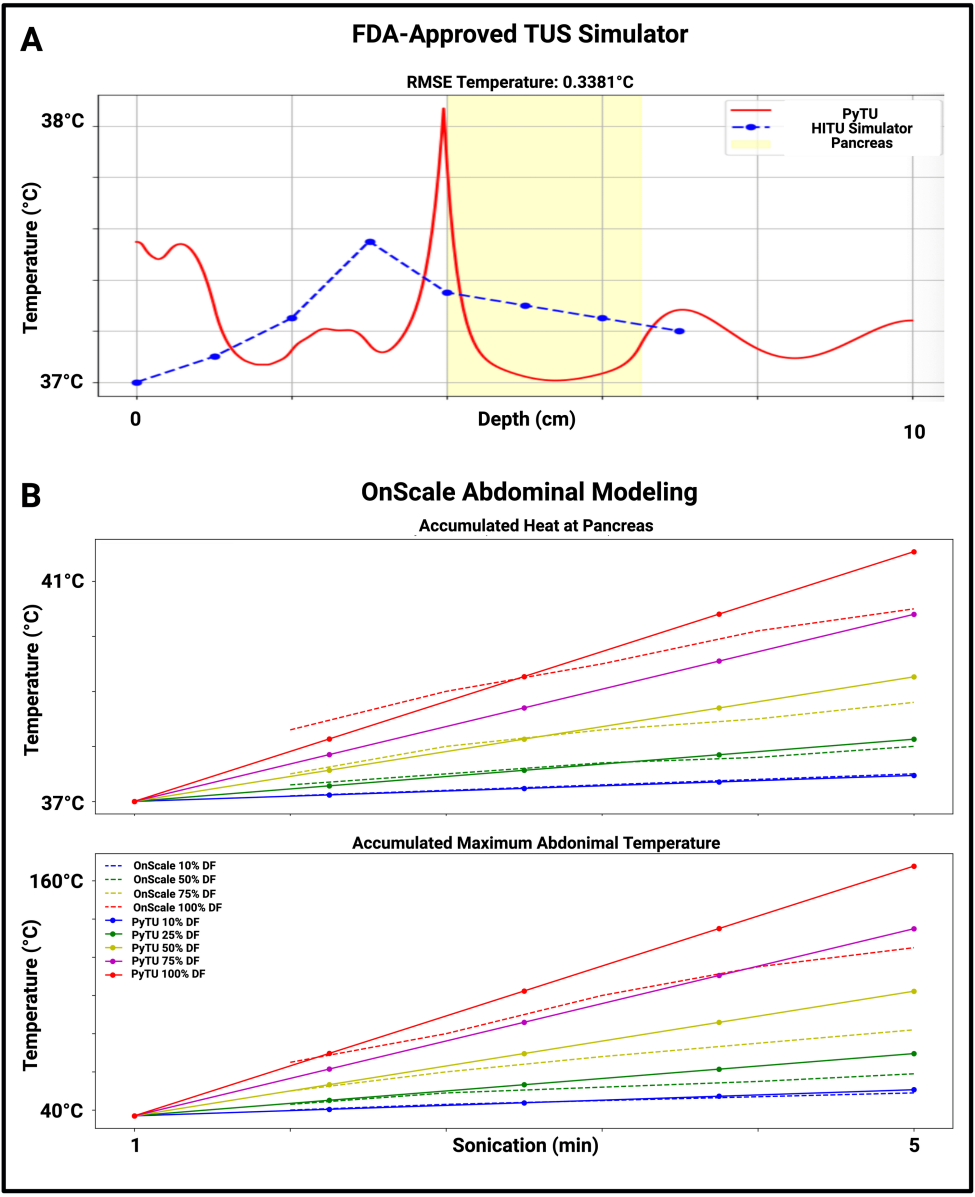
Validation of python simulations with FDA-approved HITU simulator (focused ultrasound) and pediatric therapeutic ultrasound simulations (unfocused) performed in OnScale The computational domain of the HITU simulator (focused US) is limited to 7 cm; therefore, only two out of 11 DM patients could be initially modeled with this software. Figure 7A depicts the thermal validation of the PyTU (focused US) simulation in this work, with an RMSE for temperature serving as a validation metric for a seven-year-old female DM patient. Figure 7B depicts the thermal validation of the PyTU simulation with prior OnScale (unfocused US) modeling of a 15-year-old female without DM. The thermal curves over time for each sonication and intensity parameter are included to show the similarity between the Python and OnScale models. PyTU’s focused transducer delivers more heat to the abdominal space than OnScale’s unfocused transducer, but at duty factors of less than 75%, PyTU’s thermal effects closely follow and validate OnScale’s thermal effects. PyTU is the in-house therapeutic modeling package developed in Python. All validation simulations were modeled at 1 MHz at 5 W/cm^2^ FDA: Food and Drug Administration; HITU: high-intensity therapeutic ultrasound; RMSE: root mean square error

Figure 7 incorporates multiple models with imaging review to inform potential TUS models for clinical use. Figure 7A illustrates the comparative thermal effects of focused ultrasonic stimulation of the pancreas via HITU simulator (MATLAB, 2025a) against our in-house PyTU (Python v3, 2025). Figure 7B demonstrates the comparative unfocused thermal effects of previous OnScale modeling [14] and our in-house PyTU modeling. Previous OnScale models demonstrated the feasibility of unfocused ultrasound stimulation of insulin release; however, focused ultrasound stimulation was prioritized in this work for its improved pancreatic targeting. Table 3 contains all modeling parameters used in this work. From our modeling, a frequency of 1MHz at 5 W/cm^2^ is safe and effective in the pancreatic abdomen in both focused and unfocused transducer scenarios. However, Figure 7B highlights that the dissipated heat in the focused transducer scenario delivers more heat at 100% duty factor when compared to unfocused sonication with the same US protocol. As confirmed by prior modeling [14], duty factors of 75% or greater should be avoided for safe and effective TUS for insulin release.

**Table 3:** Tissue-specific parameters used in HITU simulator/PyTU/OnScale Each US image reviewed showed each of these tissue types as intersections on the acoustic beam path. For accurate modeling, tissue properties from previous literature were used in all three simulations. Table 3 contains the sonication parameters tested in each of the models in this work. Transducer type is identified for each model, as OnScale used an unfocused transducer, HITU simulator used a focused transducer, and this study's version of PyTU utilized a focused transducer for more targeted pediatric therapeutic efficacy HITU: high-intensity therapeutic; US: ultrasound

Tissue	Speed of sound (m/s)	Density (kg/m^3^)	Attenuation (dB/cm)	Specific heat (J/kg/K)
Skin [14]	1624	1109	3.5	3391
Blood [14]	1578	1050	0.21	3617
Pancreas [14]	1591	1087	0.829	3164
Muscle [14]	1588.4	1090	0.7	3421
Bowel [32]	1560	1000	0.002	4200
Parameter	Value(s)	Model	Transducer type	
Frequency	1 MHz			
Intensities	1 W/cm^2^, 5 W/cm^2^, 10 W/cm^2^	HITU simulator	Focused (2 - 4 cm), 1.5 cm active diameter	
Duty cycle	10%, 25%, 50%, 75%, 100%	OnScale	Unfocused, 1.5 cm active diameter	
Sonication Time	1 min, 3 min, 5 min	PyTU	Focused (2 - 8 cm), 1.5 cm active diameter	

## Discussion

Review of pancreatic ultrasound in a pediatric diabetes mellitus dataset

This work’s primary contribution is a pancreatic imaging repository of the pediatric pancreas stratified by diabetic status. Upon review of our pediatric dataset (Figure 3), the pancreatic body was consistently visualized across patients, with the body visible in 95.45% of the B-mode ultrasound images. In our pediatric population, the head was visualized with less frequency (31.82%) compared to the tail (47.73%). These results suggest that the pancreatic body is the most promising initial target for pediatric TUS towards insulin release.

Prior literature examining the adult DM pancreas with MRI, CT, and US imaging revealed imaging features, such as a lack of homogeneity of pancreatic tissue, that were distinctive between DM and non-DM pancreatic tissue [5-8]. Similar imaging biomarkers, such as a decrease in pancreatic volume, have been documented with MRI imaging in pediatric patients with DM [12]. Figure 2 highlights the difficulty in distinguishing between DM and non-DM pancreatic tissue. To a trained radiologist’s review, these two pancreatic images were not identifiable as either DM or non-DM. Therefore, a more detailed analysis at the level of image texture was required to ascertain differences between DM and non-DM pancreatic tissue on US images.

Imaging feature measurements stratified by diabetic and non-diabetic patients

The analysis of US imaging features in Figures 4A-4B suggests that entropy, a measure of tissue disorder, is relatively higher in non-DM tissue when analyzing our B-mode ultrasound images. As previously mentioned, US and pixel-level relationships are influenced by speckling and noise, two properties that could fundamentally change the analysis of entropy-based features within diseased tissue contexts. While more imaging review is necessary to confirm such a statement, initial review of our dataset introduces a paradoxical finding: DM B-mode pancreatic US exhibits lower levels of entropy and higher levels of textural uniformity compared to non-DM pancreatic tissue.

The boxplots in Figure 2B display the feature of Area as (p<0.001) greater in non-DM pancreases than DM pancreases, confirming the prior observation of overall pancreatic size decrease outlined in Figure 1 [7]. Upon generation of the plots for Figures 4A-4B, the area greatly skewed the results. As such, Area was omitted for better statistical comparison of the features greater in non-DM pancreatic tissue. Visualized in Table 2, each of the 20 features analyzed showed statistically significant differences between diabetic and non-diabetic patients (p<0.05, df = 46). Features such as Centroid Y and Entropy exhibited high t-values and low p-values, suggesting a robust statistical separation between diabetic and non-diabetic images. Textural metrics measured via Haralick features (sum entropy, correlation, information measure of correlation) and local binary patterns (LBP) also exhibited a robust statistical separation between patient stratifications, further supporting the hypothesis that image features and structural complexity differ between diabetic and non-diabetic pancreatic ultrasound B-mode images.

Imaging feature correlation with diabetes mellitus

Statistical comparison via Pearson’s coefficient, t-tests, and Mutual Information scores elucidated linear and nonlinear relationships within our dataset. From Figure 5A, the dominance of Haralick features underscores their clinical relevance as established textural biomarkers. Haralick features, derived from GLCM, have been extensively validated for characterizing tissue heterogeneity [22]. The negative correlation of features like Haralick entropy, Haralick sum entropy, and entropy with DM status suggests that while disorder-based features are correlated with DM status, the disorder of pancreatic tissue is higher in non-DM patients within our dataset. This relationship could potentially be explained by pathological fibrosis and fatty tissue replacement, indicative of the diabetic pancreas [8], two pathological transformations that can create increased regions of tissue uniformity. Additionally, as pancreatic beta cells begin to atrophy during DM, the decreased size of the Islets of Langerhans in the pancreas may result in a more homogenous pancreatic tissue before more severe atrophy [33].

Imaging feature significance and mutual significance with diabetes mellitus

Looking at feature significance, Figure 5B illustrates which features were most significantly informing the label of DM status. Multiple Haralick texture features demonstrated strong statistical significance, including Haralick information measurement correlation 2, Haralick sum average, Haralick difference entropy, Haralick inverse difference moment, and Haralick entropy, all receiving -log10(p-value) scores exceeding conventional significance thresholds. While some features demonstrated both strong correlation and high significance, such as Centroid Y and Entropy, other features displayed differing patterns across statistical comparisons. For example, features such as Haralick angular second moment and LBP energy display moderate correlations in Figure 5A but display substantial significance in Figure 5B. This divergence highlights a fundamental statistical principle: correlation magnitude and statistical significance measure different aspects of data relationships.

Measurements of MI shed light on the nonlinear relationships present within the features extracted from DM and non-DM pediatric pancreatic images. Textural features such as Haralick entropy and LBP entropy (Figure 5C), which reflect heterogeneity in pixel relationships and local contrast, were consistently informative, supporting the hypothesis that imaging features affecting homogeneity of tissue are highly influential in DM pathological progression [12]. Additionally, Centroid Y, a spatial descriptor of pancreatic depth in the abdominal space, may reflect subtle anatomical shifts or size reductions in the diabetic pancreas, a phenomenon previously reported in MRI, CT, and US [5,6] [11]. Further, the strong performance of Entropy (MI = 0.246) reinforces established imaging feature principles linking tissue disorder to disease states. The relatively poor performance of traditional contrast features such as Haralick contrast (MI = 0.002) and Haralick correlation (MI = 0.005) in mutual information analysis suggests that simple intensity differences may not capture the fundamental information patterns that distinguish DM and non-DM tissues. These findings challenge conventional texture analysis approaches that rely heavily on contrast-based measures [23].

Imaging feature logistic regression

Logistic regression (univariate) analysis suggested that certain texture-uniformity measures derived from pediatric pancreatic ultrasound images, such as LBP energy and Haralick-derived GLCM metrics, contain discriminatory capacity for identifying DM status. The strong performance of these features, as stated previously, could be explained by diabetic fibrosis and fat infiltration [33], which would increase pancreatic tissue homogeneity. The narrow, bootstrapped confidence intervals further support the reproducibility of these findings within a potentially larger dataset as more patient images are acquired. Conversely, entropy-based and positional features, while statistically significant in group comparisons, exhibited low individual predictive value, underscoring the importance of evaluating both significance and predictive performance when evaluating imaging features. The combined statistical-predictive framework deployed in this work highlights the need to evaluate clinical predictive capacity beyond descriptive statistics for the selection of potential imaging biomarkers. While bootstrapping provides an estimate of the variability in AUC values, the relatively small sample size in this study limits the precision of these estimates and underscores the need for validation in larger, independent cohorts.

Overall, this multimodal feature analysis framework strengthens the evidence that ultrasound imaging feature analysis can non-invasively differentiate between diabetic and non-diabetic pancreatic tissues, supporting feature analysis integration into diagnostic and therapeutic protocols. The significance of basic morphological features (Centroid Y, Area) confirms previous studies demonstrating reduced pancreatic dimensions in DM pancreases observed in US [13]. The inclusion of textural features such as local binary patterns (LBP) and SIFT Keypoints suggests that multiscale texture analysis can capture additional discriminative information beyond traditional morphological and Haralick features. This texture analysis approach provides quantitative imaging features that may serve as objective, clinical indicators of pediatric pancreatic pathology in DM.

Deployment of multiple therapeutic ultrasound models for focused and unfocused modeling of insulin release

To further expand the TUS toolbox for DM therapy, the B-mode US images in this work informed preliminary TUS simulations. Figure 7A shows the validation of a more amenable Python software created in-house against the HITU simulator, PyTU. With a root mean square error (RMSE) of less than 0.4°C, the in-house PyTU software closely matched the focused, TUS thermal effects of FDA’s HITU simulator. Figure 7B illustrates thermal effects at the pancreas and throughout the overall abdomen across OnScale’s [14] unfocused transducer and PyTU’s focused transducer. PyTU closely followed the thermal effects of previous OnScale, unfocused TUS modeling; however, at duty factors of 100%, PyTU’s thermal effects far exceeded (55°C) those simulated in OnScale. Other than the 100% duty factor, each duty factor and intensity’s heating curve closely followed the curve suggested by OnScale. Dermal fat and muscle tissue exhibited a higher delivered temperature than pancreatic tissue by approximately 7.5%. All sonication parameters tested below 100% duty factor did not suggest deleterious thermal doses of CEM43 (<240 CEM43, maximum abdominal temperature <135°C, maximum pancreatic temperature <40°C), the clinical measure for safety in TUS [30]. By directly comparing the thermal results of PyTU to previously validated TUS software packages, this work suggests that various transducer types and software packages can be deployed in TUS modeling for pancreatic tissue treatment in a manner that is informed by ultrasound images of a patient’s abdomen.

Limitations

The primary limitation of this work is the lack of available diabetic pancreatic ultrasound imaging. While the number of patients included in this work's repository was only 22 (90 images), statistical evaluation was still able to elucidate differential imaging features between DM and non-DM pancreatic tissue. The relationships explored in this work require a larger sample size of patient images to draw clinical conclusions. However, as evidenced by the preliminary TUS modeling, this work provides preliminary support and validation for the continued exploration of ultrasound as both a diagnostic and therapeutic tool in the pediatric clinical space.

Sonographer acquisition of these images occurred on different days, with different patients, and under different imaging conditions. We acknowledge the potential impact of such variables on the resulting B-mode ultrasound image fidelity. Additionally, as the review of ultrasound images in this work was retrospective, there was no way to ‘probe’ for more pancreatic scans. Table 1 outlines the distribution of the data set for this work, providing the boundary conditions, limitations, and potential bias within the imaging dataset.

Future research directions

This initial study shows that quantitative ultrasound features can differentiate between diabetic and non-diabetic pancreatic tissue, but the limited sample size constrains further generalizability. Larger, multi-center datasets with diverse acquisition protocols will further confirm predictive robustness. Validation from a larger sampling dataset will be necessary to verify the predictive performances outlined in this work. Longitudinal studies should determine whether these imaging features appropriately reflect disease progression or TUS treatment response, supporting their potential use as reproducible, clinically relevant biomarkers for pediatric DM.

## Conclusions

We created an ultrasound imaging repository of the pediatric pancreas for image feature analysis and patient-specific TUS modeling. Safe, cost-effective, and accessible in pediatric contexts, US can both diagnose and treat pediatric patients with DM. Statistical comparison between size, texture, and morphology features elucidated linear and nonlinear relationships between B-mode pixel values and diabetic status. B-mode pixel comparison offers potential image-based biomarkers that can differentiate diabetic pancreatic tissue in future pediatric patient treatment. Analysis of pancreatic depth, entropy, and GLCM correlation (tissue homogeneity) showed quantifiable differences between DM and non-DM pediatric US images. Visual differences between DM and non-DM images were imperceivable to trained radiologists, suggesting that the diagnostic utility of pancreatic US should be further investigated. To expand the clinical utility of DM therapy, patient-specific models incorporating tissue-specific TUS simulations in both focused and unfocused stimulation were surveyed for potential insulin release in diabetic models. This work contributes to referential US imaging features and preliminary modeling of patient-specific TUS DM treatment.
